# Survival Mechanisms of *Campylobacter hepaticus* Identified by Genomic Analysis and Comparative Transcriptomic Analysis of *in vivo* and *in vitro* Derived Bacteria

**DOI:** 10.3389/fmicb.2019.00107

**Published:** 2019-02-05

**Authors:** Thi Thu Hao Van, Jake A. Lacey, Ben Vezina, Canh Phung, Arif Anwar, Peter C. Scott, Robert J. Moore

**Affiliations:** ^1^School of Science, RMIT University, Bundoora, VIC, Australia; ^2^Doherty Department, University of Melbourne at the Peter Doherty Institute for Infection and Immunity, Melbourne, VIC, Australia; ^3^Scolexia Pty Ltd., Moonee Ponds, VIC, Australia

**Keywords:** comparative genomics, transcriptomics, *Campylobacter hepaticus*, glucose utilization, polyhydroxybutyrate, stress response

## Abstract

Chickens infected with *Campylobacter jejuni* or *Campylobacter coli* are largely asymptomatic, however, infection with the closely related species, *Campylobacter hepaticus*, can result in Spotty Liver Disease (SLD). *C. hepaticus* has been detected in the liver, bile, small intestine and caecum of SLD affected chickens. The survival and colonization mechanisms that *C. hepaticus* uses to colonize chickens remain unknown. In this study, we compared the genome sequences of 14 newly sequenced Australian isolates of *C. hepaticus*, isolates from outbreaks in the United Kingdom, and reference strains of *C. jejuni* and *C. coli*, with the aim of identifying virulence genes associated with SLD. We also carried out global comparative transcriptomic analysis between *C. hepaticus* recovered from the bile of SLD infected chickens and *C. hepaticus* grown *in vitro*. This revealed how the bacteria adapt to proliferate in the challenging host environment in which they are found. Additionally, biochemical experiments confirmed some *in silico* metabolic predictions. We found that, unlike other *Campylobacter* sp., *C. hepaticus* encodes glucose and polyhydroxybutyrate metabolism pathways. This study demonstrated the metabolic plasticity of *C. hepaticus*, which may contribute to survival in the competitive, nutrient and energy-limited environment of the chicken. Transcriptomic analysis indicated that gene clusters associated with glucose utilization, stress response, hydrogen metabolism, and sialic acid modification may play an important role in the pathogenicity of *C. hepaticus*. An understanding of the survival and virulence mechanisms that *C. hepaticus* uses will help to direct the development of effective intervention methods to protect birds from the debilitating effects of SLD.

## Introduction

Spotty Liver Disease (SLD) causes significant egg production losses and mortality in layer birds (Crawshaw and Young, 2003; Grimes and Reece, 2011). It has been sporadically reported over the last 60 years, first from the United States then from Canada, New Zealand, Estonia, the United Kingdom, Austria, Germany and Australia (Tudor, 1954; Bertschinger, 1965; Leesment and Parve, 1965; Truscott and Stockdale, 1966; Kölbl and Willinger, 1967; Pohl et al., 1969; Crawshaw and Irvine, 2012). The disease has become increasingly common in Australia over the last decade and is now considered one of the most significant health challenges in the egg industry (Grimes and Reece, 2011). However, it was only in 2015 that a novel *Campylobacter* species was isolated from SLD cases in the UK and in 2016 *Campylobacter hepaticus* was identified and characterized from Australian cases of SLD (Crawshaw et al., 2015; Van et al., 2016). In 2017 *C. hepaticus* was definitively shown to be the cause of SLD (Van et al., 2016, 2017a,b).

As *C. hepaticus* has only recently been identified, the study of its biology is just beginning. The draft genomes of the type strain, *C. hepaticus* HV10, isolated from the liver of an Australian SLD affected chicken, and a series of British isolates are available (Van et al., 2016; Petrovska et al., 2017). *C. hepaticus* is most closely related to the foodborne pathogens *C. jejuni* and *C. coli*. However, *C. hepaticus* lacks some of the well-identified virulence genes found in *C. jejuni*, such as the cytolethal distending toxin (CDT) genes. It is anticipated that *C. hepaticus* must harbor a set of genes responsible for the pathogenesis observed in SLD affected chickens. These genes must encode products that lead to damage to the liver, as well as mortality and egg production losses. *C. hepaticus* has been isolated from liver and bile of SLD affected birds and has also been shown to be present in the gastrointestinal tract (Van et al., 2017b, 2018). Bile is a challenging environment and presumably *C. hepaticus* must orchestrate the expression of certain genes to help them survive within this niche.

High-throughput next-generation sequencing (NGS) has revolutionized transcriptomics by allowing global expression studies through RNA sequencing or RNA-Seq, through the sequencing of complementary DNA (cDNA) (Kukurba and Montgomery, 2015). RNA-Seq has rapidly taken the place of previous methods of genome-wide quantification of gene expression (transcriptomics) including hybridization-based microarrays and Sanger sequencing-based approaches. RNA-Seq has proven to be a fast, sensitive and reliable method because of the high sequence coverage. This approach has been used widely to study bacterial transcriptomics (Taveirne et al., 2013; Rao et al., 2015).

Genes that encode products required for niche adaptation, colonization, and virulence are yet to be identified in *C. hepaticus*. The aim of this study was to investigate potential virulence factors that could explain the pathogenic nature of *C. hepaticus* in poultry and identify strategies that *C. hepaticus* uses to colonize and survive in the host. We compared the genome sequences of 14 newly sequenced Australian isolates of *C. hepaticus* to nine *C. hepaticus* isolates from outbreaks in the United Kingdom that had previously been sequenced (Petrovska et al., 2017) and 10 reference strains of *C. jejuni* and *C. coli*, with the aim of identifying potential virulence genes in *C. hepaticus*. Furthermore, we studied the differential gene expression of *C. hepaticus* HV10; comparing the transcriptomes of *in vivo* (recovered from chicken bile samples) and *in vitro* (cultured on horse blood agar plates) grown bacteria. These results were combined with the comparative genomics analysis to investigate the mechanisms that *C. hepaticus* may use to adapt to the challenging bile environment and cause disease in chickens.

## Materials and Methods

### Whole Genome Analysis of *C. hepaticus* Australian and UK Isolates

*Campylobacter hepaticus* HV10 was published as a draft genome (Van et al., 2017b). In this study, the complete closed genome of HV10 was obtained by combining short Illumina reads, and long PacBio reads and a series of bioinformatics pipelines as described in Lacey et al. (2018). The closed *C. hepaticus* HV10 genome was deposited in the NCBI database (accession number: CP031611.1). Fourteen Australian *C. hepaticus* isolates, each from an independent SLD outbreak event, were sequenced (methods as described in Van et al. (2016) and compared with the publicly available whole genome sequences of nine *C. hepaticus* isolates from the United Kingdom, five *C. jejuni*, and five *C. coli* representative genomes extracted from the NCBI database (Table 1). Genomes were assembled using the A5MiSeq pipeline version 20150522 (Coil et al., 2015) and they were annotated using both Prokka 1.14-dev and RAST version 2.0 (Aziz et al., 2008; Seemann, 2014). All assemblies and read sets were deposited in NCBI (Bioproject PRJNA485661).

**Table 1:** Isolates used in this study.

**Organism_name**	**Isolates**	**Number of proteins (Prokka)**	**GC%**	**Genome size**	**Origin**	**Accession number**
*Campylobacter hepaticus*	HV10	1498	28.2	1520669	VIC-Australia	NZ_CP031611.1
*Campylobacter hepaticus*	HV16	1492	28.2	1482877	VIC-Australia	QURU00000000
*Campylobacter hepaticus*	27L	1549	28.1	1530133	VIC-Australia	QUSC00000000
*Campylobacter hepaticus*	68B	1493	28.2	1484116	VIC-Australia	QUSB00000000
*Campylobacter hepaticus*	84B	1554	28.1	1531838	VIC-Australia	QURX00000000
*Campylobacter hepaticus*	ACE1	1546	28.2	1530407	VIC-Australia	QUSA00000000
*Campylobacter hepaticus*	ACE8659	1551	28.1	1532304	VIC-Australia	QURZ00000000
*Campylobacter hepaticus*	ACEM3A	1549	28.2	1535304	VIC-Australia	QURY00000000
*Campylobacter hepaticus*	DISRED	1491	28.2	1486604	VIC-Australia	QURV00000000
*Campylobacter hepaticus*	NSW44L	1497	28.2	1483699	NSW-Australia	QURM00000000
*Campylobacter hepaticus*	SA32L	1495	28.2	1484444	SA-Australia	QURT00000000
*Campylobacter hepaticus*	SA34L	1493	28.2	1481686	SA-Australia	QURS00000000
*Campylobacter hepaticus*	19L	1472	28.1	1517721	QLD-Australia	QUSD00000000
*Campylobacter hepaticus*	54L	1473	28.2	1518322	QLD-Australia	QURW00000000
*Campylobacter hepaticus*	S10-0209	1555	28.3	1520159	UK	ERR1802474
*Campylobacter hepaticus*	S11-0036	1495	28.3	1475458	UK	ERR1802475
*Campylobacter hepaticus*	S11-0069	1489	28.3	1481897	UK	ERR1802476
*Campylobacter hepaticus*	S11-0071	1490	28.2	1482032	UK	ERR1802477
*Campylobacter hepaticus*	S11-010	1602	28.3	1565372	UK	ERR1802478
*Campylobacter hepaticus*	S11-0038	1488	28.3	1476273	UK	ERR1802479
*Campylobacter hepaticus*	S11-5013	1555	28.3	1521121	UK	ERR1802480
*Campylobacter hepaticus*	S12-0322	1519	28.3	1516203	UK	ERR1802482
*Campylobacter hepaticus*	S12-1018	1595	28.3	1520158	UK	ERR1802483
*Campylobacter jejuni*	81-176	1622	30.6	1616554	-	CP000538.1
*Campylobacter jejuni*	NCTC11168	1658	30.6	1641481	-	NC_002163.1
*Campylobacter jejuni*	R14	1954	30.3	1795858	-	CP005081.1
*Campylobacter jejuni*	RM1221	1871	30.3	1777831	-	CP000025.1
*Campylobacter jejuni*	S3	1759	30.5	1681364	-	CP001960.1
*Campylobacter coli*	CVM_N29710	1699	31.5	1673221	-	CP004066.1
*Campylobacter coli*	FB1	1672	31.6	1658607	-	CP011016.1
*Campylobacter coli*	OR12	2155	30.8	2033903	-	CP019977.1
*Campylobacter coli*	RM4661	1913	31.2	1824273	-	CP007181
*Campylobacter coli*	YH501	1704	31.6	1668523	-	CP015528.1

Homolog identification and pan genome investigation of all predicted coding sequences was performed using two independent methods. Clustering using USEARCH v10.0 at 70% similarity across 70% protein length within the Bacterial Pan genomes analysis (BPGA v1.3) tool pipeline (Chaudhari et al., 2016), and with Roary v3.12 at a protein identity of 70% (-i70) and no splitting of paralogs (-s) (Page et al., 2015).

For phylogenetic inferences, single nucleotide variants (SNVs) were called by aligning reads to a reference genome, *C. hepaticus* strain HV10, using Snippy v3.2 (https://github.com/tseemann/snippy). Gubbins v2.3.4 (Croucher et al., 2015) was used for the detection and removal of recombinogenic regions, and PHASTER was used to screen for prophage integrations that would be outside the clonal frame (Zhou et al., 2011). A Maximum-likelihood tree was built in RAxML v8.2.12 (Stamatakis, 2014) using the general-time reversible model (GTRCAT) with 1,000 bootstrap replicates. Clustering of strains was performed using RAMI at a patristic distance threshold of 0.05 divergence (Pommier et al., 2009).

The map of the DNA features of *C. hepaticus* reference strain HV10 was produced in DNAplotter v16.0.0 (Carver et al., 2009). The core and pan genome plots of the 24 *C. hepaticus* isolates and COG distribution plot of functional categories for coding sequences within these 24 genomes were produced using BPGA.

### Identification of Virulence Associated Genes of *C. hepaticus*

The *C. hepaticus* HV10 genome was examined for potential virulence genes by searching against the Virulence Factor Database (http://www.mgc.ac.cn/VFs/main.htm) in ABRicate (https://github.com/tseemann/abricate) (data assessed June 2018) and by inspecting the annotated genome manually. To elucidate the genetic potential of *C. hepaticus* to cause SLD a pan genome wide association study (PGWAS) was performed using Scoary v1.6.10 (Brynildsrud et al., 2016), and each gene in the *C. hepaticus* pan genome for association to SLD was screened. Genes of interest were identified as specific to the *C. hepaticus* genomes (present in 100% of isolates and absent in all reference *C. coli* and *C. jejuni* strains). The functionality of these unique genes was inferred from matches to the Pfam database (Finn et al., 2014), Interproscan (Jones et al., 2014), Swiss-Prot (Bairoch and Apweiler, 1997) and Uniprot (UniProt Consortium, 2015). Product descriptions were assigned with homologs of 70% similarity across 90% of protein length. CRISPRFinder (v2017-05-09) (Grissa et al., 2007) was used to analyse CRISPRs.

### Investigation of *C. hepaticus* Horizontally Acquired Elements

The annotated genomes of the 23 *C. hepaticus* isolates were first manually inspected for any potential acquired genetic materials. Contigs with genes annotated as suspected plasmid elements were Blasted against the NCBI database. Significant matches were determined by matches >90% coverage and identity. ABRicate v0.8.7 was used to screen for antibiotic resistance genes.

### RNA-Seq Analysis of *C. hepaticus* During *in vivo* Colonization and *in vitro* Growth

*C. hepaticus* HV10 (Van et al., 2016) were grown on Brucella agar (Becton Dickinson) with 5% horse blood (HBA) and incubated at 37°C in microaerobic conditions using CampyGen gas packs (Oxoid).

SLD in chickens was induced by challenge with *C. hepaticus* HV10. The animal experimentation was approved by the Wildlife and Small Institutions Animal Ethics Committee of the Victorian Department of Economic Development, Jobs, Transport and Resources (approval number 14.16). Hy-Line layer hens (26-weeks old, sourced from a farm that had not observed any SLD in their flocks for several years) were used in the study. Birds were also tested for *C. hepaticus* to ensure they were *C. hepaticus* negative before the trial by using the specific PCR developed by Van et al. (2017b) on the cloacal swab samples. Experimental chickens were challenged as previously described (Van et al., 2017a). Briefly, birds were challenged by direct oral gavage with 1x10^9^ CFU of *C. hepaticus* HV10 strain in 1 ml of Brucella broth, whereas the control chickens were given 1 ml of Brucella broth. The birds were sacrificed 5 days post-challenge and the livers were examined for lesions. Bile samples from all chickens were taken aseptically from the gall bladder and placed in tubes containing RNAlater (Qiagen) for RNA isolation. Samples were kept on ice, transported to the laboratory and processed immediately.

For RNA isolation from *C. hepaticus* grown in HBA (*in vitro*), *C. hepaticus* was harvested from HBA plates and resuspended in Brucella broth to an OD_600_ of 0.5 then centrifuged. The cell pellet was resuspended in RNAlater to stabilize RNA. RNA was extracted using the ScriptSeq Complete kit (Epicenter) following the manufacturer's instructions. The RNA was treated with DNase I (NEB) to remove DNA contamination. The quality of the total RNA in the samples was checked using a Nanodrop spectrophotometer (Thermofisher). RNA was also electrophoresed on a 1% agarose gel and PCR amplified using SLD specific primers (to check genomic DNA contamination) as previously described (Van et al., 2017b). RNA concentration was measured using the Qubit RNA Assay Kit (Life Technologies). The RNA samples were stored at −80°C. Both *in vitro* and *in vivo* samples were done in triplicate.

Ribosomal RNA was first removed from the RNA samples using a Ribo-Zero Magnetic Kit (Bacteria) (Illumina). Libraries for Illumina sequencing were prepared using a ScriptSeq v2 RNA-Seq Library Preparation Kit (Illumina) from the rRNA-deleted RNA. The libraries were sequenced on an Illumina MiSeq platform using 300 bp paired end reads.

The Illumina reads were mapped to the reference genome and differentially expressed genes (DEGs) were identified. Raw reads were quality trimmed using Trimmomatic version 0.36 (Bolger et al., 2014), and the trimmed reads were aligned against the *C. hepaticus* HV10 reference genome using BWA (Li and Durbin, 2010). The SAM files were imported into Blast2Go version 4.1.9 for unique read counts and differential expression analysis (Conesa and Götz, 2008). Parameters for classifying significantly expressed genes (DEGs) were ≥2-fold differences in the transcript abundance and ≤ 0.5% false discovery rate (FDR). The list of up-regulated/down-regulated genes were motif scanned to investigate their biological significances and SEED viewer was used for subsystem functional categorization of the predicted open reading frames (ORFs) from RAST annotation. DEGs were further examined by determining the KEGG Biosynthesis pathway to which they belonged.

### Confirmation of the Glucose Utilization Ability of *C. hepaticus*

*Campylobacter jejuni* strain 81116 (NCTC11828), *C. coli* NCTC 11366 and three *C. hepaticus* isolates, *C. hepticus* HV10, *C. hepaticus* 19L and *C. hepaticus* 44L were used in glucose utilization studies. After cultures were grown in HBA for 3 days, cells were collected and resuspended in physiological saline (0.9% NaCl) to an OD_600_ of 1.0. The medium used to test the ability of *C. hepaticus* to utilize glucose consisted of inorganic salts (IS) as described previously (Alazzam et al., 2011). L-cysteine (0.2 mM) was used as a nitrogen source, and α-D-glucose (10 mM) (Sigma) was used as the sole carbon source. The experiment was carried out in 24-well plates. Each well-contained 100 μl of culture (OD_600_ = 1). Controls included culture in IS plus L-cysteine only and IS plus α-D-glucose only. 2,3,5 tetrazolium chloride (TTC) (Sigma) (0.0665 g/L) was used as an indicator in all wells (Menolasino, 1959). A color change is observed in growing cultures, indicating utilization. The color change results were read after 36 h of incubation at 37°C. *C. hepaticus, C. jejuni*, and *C. coli* were also grown in Brucella broth to confirm their viability. The experiment was repeated twice, each time in biological triplicate.

## Results

### *C. hepaticus* Has a Closed Pan Genome

By combining short Illumina reads and long PacBio reads, the complete closed genome of *C. hepaticus* HV10 strain was obtained in this study (NCBI accession number CP031611). The genome size of *C. hepaticus* HV10 is 1,520,669 bp with a GC content of 28.2%. It has been used as the reference genome to compare with the other draft genome sequences.

**Figure 1:**
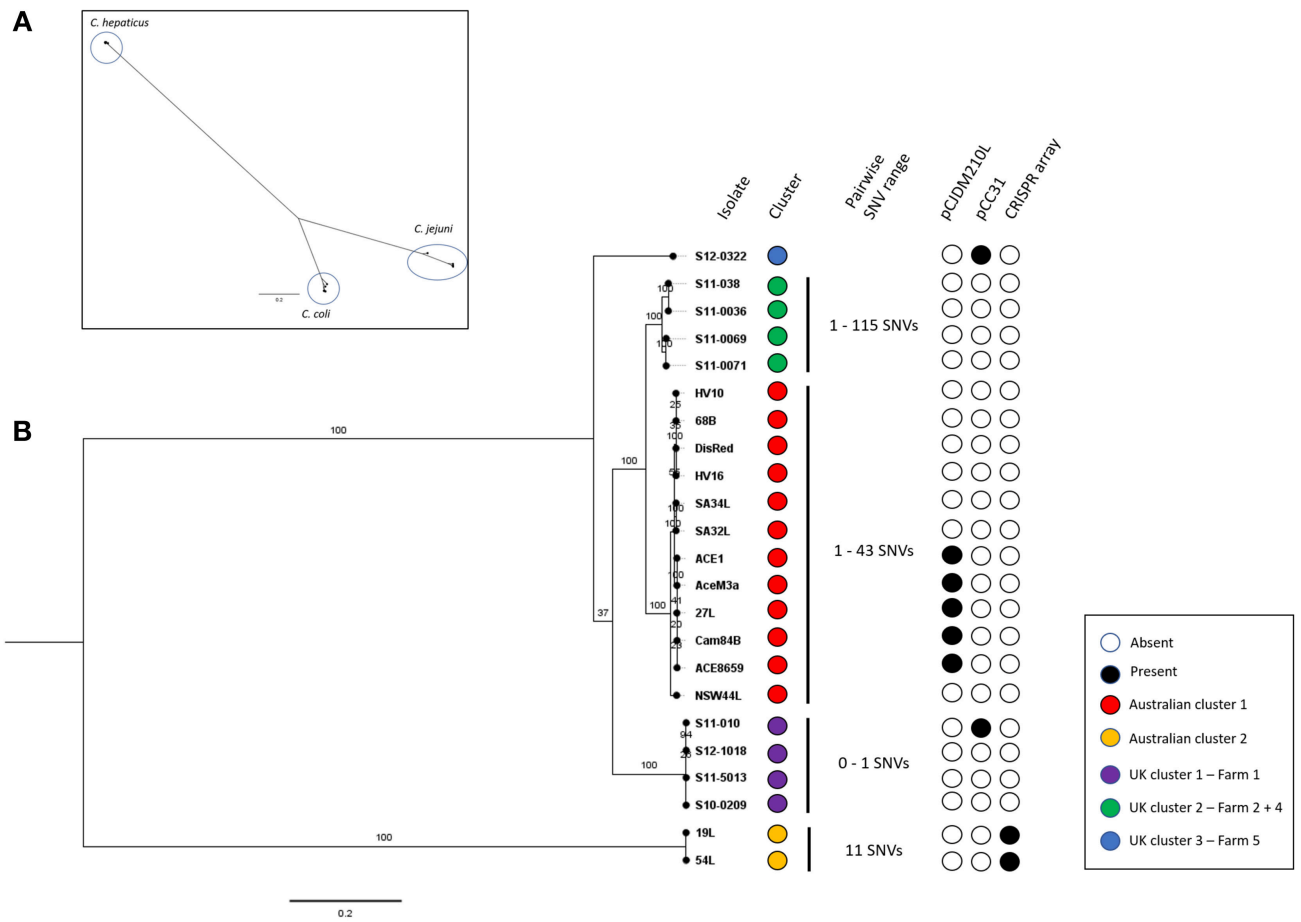
**(A)** *Campylobacter* spp. core genome phylogeny. Core genome alignment to the *C. hepaticus* reference strain HV10 of all *C. hepaticus* isolates and 5 isolates of *C. coli* and *C. jejuni* was performed. A maximum likelihood tree was inferred from 33,157 SNVs and built using RAxML using the general-reversible model (GTRCAT) with 1,000 bootstrap replicates. Scale bar shows nucleotide divergence. Three clusters corresponding to each of the species were circled. **(B)** *C. hepaticus* core genome phylogeny of Australian and UK isolates against the HV10 reference strain. Maximum-likelihood tree was inferred from 4,812 SNVs and built in RAxML using the general-reversible model (GTRCAT) with 1,000 bootstrap replicates. Scale bar shows nucleotide divergence. Clustering was performed using RAMI at a patristic distance threshold of 0.05, corresponding phylogroups were colored blue, green, red, purple, and yellow. The range of pairwise SNVs within each phylogroup is shown (minimum value—maximum value). The presence key accessory genome elements including the CRISPR-array and the two plasmids pCC31 and pCJDM210L are shown.

Genome sizes of the *C. hepaticus* isolates ranged from 1.475 to 1.565 Mb whereas the representative *C. jejuni* genomes ranged between 1.617 and 1.800 Mb and *C. coli* genome sizes ranged between 1.668 and 2.034 Mb. There were between 1472 and 1595 annotated protein-coding genes predicted to be encoded by the 23 *C. hepaticus* isolates, whereas that were 1622–1954 for five *C. jejuni* isolates and 1672–2155 for five *C. coli* isolates (Table 1). A total of 1,059 core genes were conserved in all 33 genomes (fourteen Australian *C. hepaticus* isolates, nine United Kingdom *C. hepaticus* isolates, five *C. jejuni* and five *C. coli*). Maximum likelihood (ML) phylogeny as produced from RAxML was inferred from 33,157 SNVs. The core genome tree of the 33 genomes comprised 3 phylogenetically distinct lineages corresponding to each of the 3 species (Figure 1A).

Within each phylogroup the mean pairwise nucleotide divergence between genomes was ~3.58%, but the *C. hepaticu*s genomes showed much less divergence (0.46%) than the *C. coli* (6%) and *C. jejuni* (4.29%) genomes. Nucleotide divergence from *C. hepaticus* to *C. coli* and *C. jejuni* was ~68 and 63.8%, respectively (calculated across core sequence alignments). The nucleotide divergence between *C. coli* and *C. jejuni* was 23.16%, indicating that *C. coli* and *C. jejuni* are more closely related to each other than to *C. hepaticus*. Gubbins did not detect any recombinogenic regions, showing there is no evidence of recombination between the phylogroups, adding further support to the clear separation of *C. hepaticus* from *C. jejuni* and *C. coli*. Accessory genome variation also supports the separation of *C. hepaticus* from the other species, demonstrating that each phylogroup is a discrete bacterial population that is evolving independently, with limited homologous recombination between groups.

Comparison of the genome sequences of the 23 *C*. *hepaticus* isolates revealed a total of 1,360 core genes conserved across all genomes. Maximum Likelihood (ML) phylogeny was produced from 4,812 core genome SNVs and revealed a shallow branching population structure with high bootstrapping support. There is a high level of conservation within the genomes (median of 95.19% coverage of the reference strain HV10), possibly due to the specific niche adaption of the species. The *C. hepaticus* genomes clustered into five phylogenetic lineages based on the core genome ML tree using a patristic distance of 0.05 (Figure 1B).

The Australian isolates formed two lineages, which differed by on average ~4,429 SNPs. UK isolates formed three lineages. The Australian HV10 phylogroup differ from the UK isolates by between ~500 and 1300 SNPs. The UK lineages, represented by UK cluster 1 and UK cluster 2, differ by ~1,100 SNPs while UK lineage 3 (single isolate) differs to the rest of the UK isolates by ~1,100 to ~1,300 SNPs. Within each phylogroup the SNP frequency was very low with an average of ~41 SNPs (range 0–115 SNPs).

**Figure 2:**
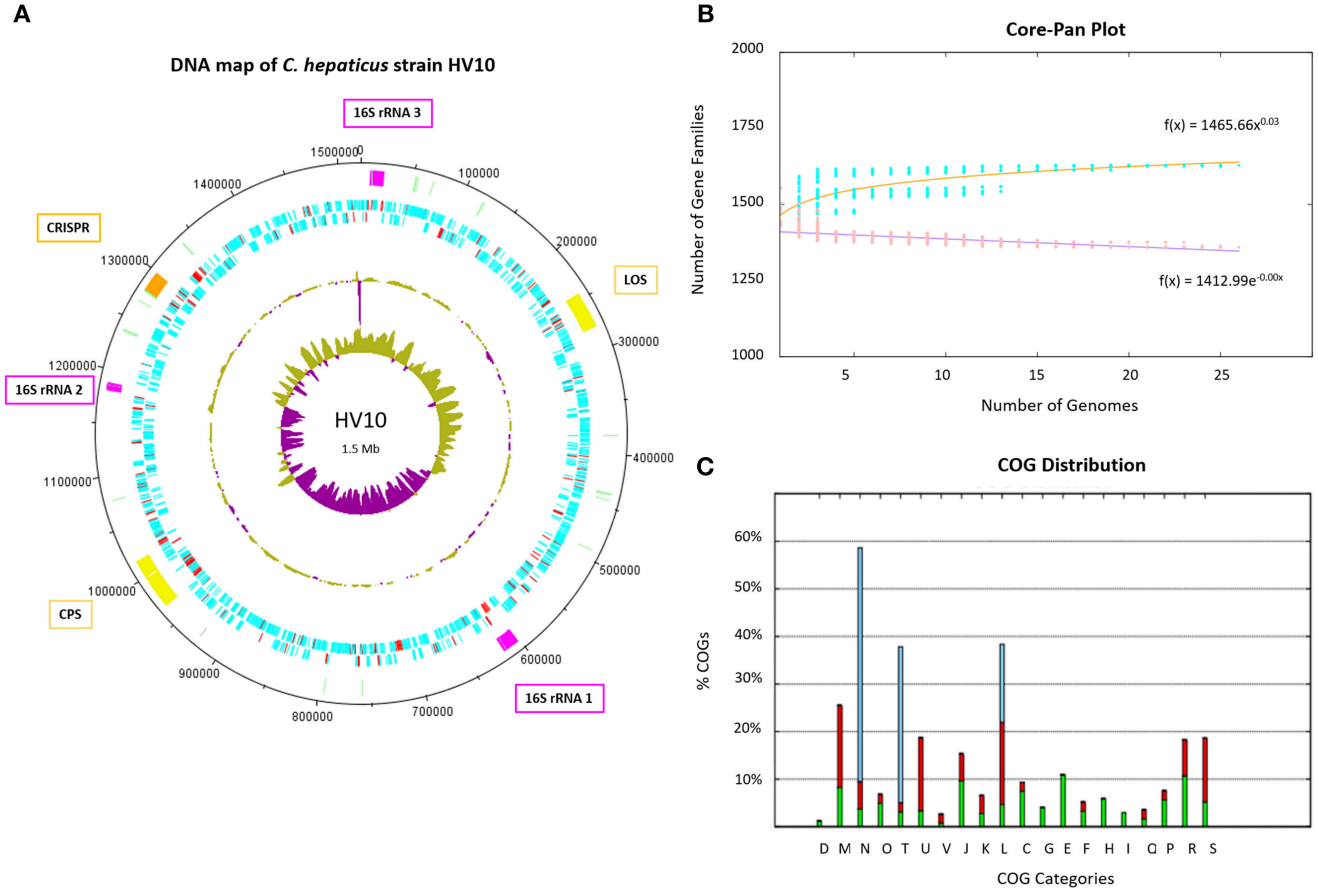
*C. hepaticus* genomes. **(A)** DNA map of the *C. hepaticus* reference strain HV10 produced in DNAplotter (Carver et al., 2009). From the most inner to outer rings shows: GC-skew, GC-content, Coding sequence (CDS) on reverse strains, CDS on forward strand, and key loci of interest. The CDS are colored blue and red, where red unique to *C. hepaticus* but not present in *C. jejuni* or *C. coli*. The loci of interest are colored as follows; pink for the three 16S ribosomal RNA operons including gene insertions, yellow for the lipooligosaccharide (LOS) and capsule (CPS) biosynthesis loci, pale green for tRNA, and orange for the CRISPR-cas machinery including gene insertions. Major ticks are observed at every 100 kb and minor ticks at 50 kb. The ribosomal operon numbering refers to the variants shown in Figure 3. **(B)** Core and pan genome plot of the 24 *C. hepaticus* genomes. The gene accumulation curves revealed an almost closed pan genome for the species. **(C)** COG distribution plot of functional categories for coding sequences within the 24 *C. hepaticus* genomes. Green represents core genes, red accessory genes and blue unique genes. X-axis letters; (D) Cell cycle control, (M) Cell wall biogenesis, (N) cell motility, (O) Post-translational modification, (T) Signal transduction, (U) Intracellular trafficking, (V) defense mechanisms, (J) Translation, (K) Transcription, (L) Replication, (C) Energy production, (G) Carbohydrate transport and metabolism, (E) Amino acid transport and metabolism, (F) Nucleotide transport and metabolism, (H) Coenzyme transport and metabolism, (I) lipid transport and metabolism, (Q) Secondary metabolites, (P) inorganic ion transport and metabolism, (R) General function and (S) function unknown. It can be observed transport and metabolism categories of various substrates are mostly encoded in the core genome (G and E), while most of the accessory and unique genome variation is categorized as replication (L), signal transduction (T) cell wall biogenesis (M), and motility (N).

A pan genome of 1,709 unique protein-coding sequences was identified across the 23 *C. hepaticus* isolates. A map of *C. hepaticus* HV10 DNA features is presented in Figure 2A. The rapid plateauing of the gene accumulation curve (Figure 2B) revealed an almost closed pan genome, suggesting most of the genetic diversity has been discovered, despite the relatively small sample size of sequenced genomes. Each genome carried on average 103 accessory genes (range = 70–192), and most of those genes were associated with multiple strains, with very few rare/unique (single isolate) accessory genes. This supports the hypothesis of genome reduction/speciation events in *C. hepaticus* as suggested by Petrovska et al. (2017). Isolates with >100 accessory genes were found to have sequences associated with mobilizable tetracycline resistance plasmids, highly similar to *Campylobacter* plasmids pCJDM210L and pCC31. The COG distribution plot of functional categories for coding sequences within the 24 *C. hepaticus* genomes (Figure 2C) showed that transport and metabolism categories of various substrates are mostly encoded in the core genome, while most of the accessory and unique genome variation is categorized as replication, signal transduction, cell wall biogenesis, and motility.

### Genetic Determinants of *C. hepaticus*—Niche Adaption and Virulence

Initial annotation and characterization of the *C. hepaticus* isolates, using the type strain HV10 as the reference sequence for the species, showed the typical structure for a *Campylobacter* genome with many genes encoding chemotaxis (11 genes), motility (47 genes), adherence/surface protein (59 genes), as well as various metabolism loci for acquisition of metals and carbohydrates (Table 2).

**Table 2:** Putative virulence genes and the changes in gene regulation *in vivo* compared to *in vitro*.

**Virulence Factor**	**Gene**	**Function**	**Locus tag (HV10)**	**Genes up/down -regulated *in vivo* compared to *in vitro/*logFC*;* no changes (–)**
Chemotaxis	*cheA*	Sensor histidine kinase	HV10_01436	Up/1.19
	*cheB*	Signal transduction response regulator	HV10_00461	Down/−1.63
	*cheR*	Methyl transferase	HV10_00462	Down/−1.70
	*cheV*	Methyl coupling protein	HV10_01437	–
	*cheW*	Methyl coupling protein	HV10_01435	Up/1.04
	*cheY*	Methyl coupling protein–flagella *fliM* associated	HV10_00280	–
	–	Chemotaxis protein–“97% to jejuni”^*^	HV10_00033	–
	–	Chemotaxis protein–“86% to jejuni”^*^	HV10_00652	Up/1.42
	–	Chemotaxis protein–“83% to coli”^*^	HV10_01414	–
	–	Methyl–accepting chemotaxis protein–“87% to jejuni”^*^	HV10_00814	Down/−1.43
	–	Methyl-accepting chemotaxis protein–“81% to jejuni”^*^	HV10_00844	Up/1.38
Motility	*motA*	Flagella motor protein	HV10_01480	Up/1.22
	*motB*	Flagella motor protein	HV10_01479	–
	*fliA*	RNA polymerase sigma factor	HV10_01172	
	*fliE*	Flagella hook-basal body complex protein	HV10_00910	–
	*fliF*	Flagella M-ring protein	HV10_01460	–
	*fliG*	Flagella motor switch protein	HV10_01461	–
	*fliH*	Flagella assembly protein	HV10_01462	–
	*fliI*	ATPase	HV10_01055	–
	*fliK*	Flagella hook-length control protein	HV10_01180	–
	*fliL*	Flagella basal body protein	HV10_00971	–
	*fliM*	Flagella motor switch protein	HV10_01173	–
	*fliN*	Flagella motor switch protein	HV10_01491	Up/1.26
	*fliP*	Flagella biosynthetic protein	HV10_00545	–
	*fliQ*	Flagella biosynthetic protein	HV10_01287	–
	*fliR*	Flagella biosynthetic protein	HV10_00219	Down/−0.88
	*fliS*	Flagella export chaperone	HV10_00887	–
	*fliW*	Flagella assembly factor	HV10_00325	–
	*fliY*	Flagella motor switch protein	HV10_01174	–
	*flgA*	Flagella basal body P-ring formation	HV10_00587	–
	*flgB*	Flagella basal body rod	HV10_00908	Down/−1.59
	*flgC*	Flagella basal body rod	HV10_00909	–
	*flgE*	Flagella hook protein	HV10_01178	Down/−1.57
	*flgE*	Flagella hook protein	HV10_01221	Down/−1.26
	*flgG*	Flagella basal body rod	HV10_00654-00655	Down/−2.00–3.03
	*flgI*	Flagella biosynthesis protein	HV10_01034	Down/−1.57
	*flgK*	Flagella hook-length control protein	HV10_01180	–
	*flgL*	Flagellin biosynthesis protein	HV10_00496	–
	*flgN*	Flagella protein	HV10_01037	–
	*flgK*	Flagella hook-associated protein	HV10_01038	–
	*flgP*	Lipoprotein required for motility	HV10_373	Down/−2.34
	*flgQ*	Protein required for motility	HV10_374	Down/−2.65
	*flgR*	Signal-transduction regulatory protein FlgR	HV10_375	–
	*flgS*	Sensor histidine kinase	HV10_565	–
	*flgH*	Flagella L-ring prptein	HV10_664	Down/−2.46
	*flhA*	Flagella biosynthesis protein	HV10_00500	–
	*flhB*	Flagella biosynthesis protein	HV10_01478	–
	*flhF*	Flagella biosynthesis protein	HV10_01169	–
	*flhG*	Flagellr synthesis regulator	HV10_01170	–
	*flaB*	Flagellin subunit protein	HV10_00091	Down/−2.30
	*flaG*	Flagella biosynthesis protein	HV10_00889	–
	*flgD*	Flagella basal body rod modification	HV10_01179	Down/−1.65
	*fliD*	Flagella capping protein	HV10_00888	–
	*fliC*	Flagellin	HV10_00090	–
	*fliQ*	Flagella biosynthesis protein FliQ	HV10_01278	–
	*pflA*	Paralyzed flagella protein PflA	HV10_01413	–
	*rpoN*	RNA polymerase sigma-54 factor	HV10_00672	–
	*eptC*	Phosphoethanolamine lipid A transferase	HV10_00849	–
Adherence	*DnaJ*	Molecular chaperone	HV10_00142	–
	-	Adhesion/export protein	HV10_00448	–
	-	Hemagglutinin	HV10_01341	Down/−2.89
	*pebA*	Adhesin	HV10_00464	–
	ciaB	Campylobacter invasion antigen B	HV10_470	–
Iron uptake	-	Ferritin	HV10_00733	–
	*feoB*			
	*hugZ*	Heme oxygenase	HV10_01365	Down/−2.00
	NapG	Ferredoxin-type protein	HV10_00577	Up/2.25
	*glcG*	Heme-binding protein	HV10_01392	–
Copper	-	Copper chaperone	HV10_00232	–
	-	Copper-translocating P-type ATPase	HV10_00233	Up/1.09
	-	laccase	HV10_00178	–
Type II Secretion system (transformation)	*gspF*	General secretion pathway protein	HV10_00776	Down/−1.75
	*cstE/gspE*	Type II/IV Secretion system	HV10_00777	–
	-	Transformation system protein	HV10_00778	–
	-	Transformation system protein	HV10_00779	Down/−2.45
	*mshL/ctsX/gspD*	Pilus biogenesis protein	HV10_00780	–
	-	Transformation system protein	HV10_00781	–
	-	Pyruvate: ferredoxin	HV10_00782	–
	-	HAD family hydrolase	HV10_00783	–
	cadF	Outer membrane fibronectin-binding protein	HV10_00784	–
Glucose utilization^a^	-	Glucose/galactose MFS transporter^a^	HV10_00601	–
	*pgi*	Glucose-6-phosphate isomerase^a^	HV10_00602	–
	*glK*	Glucokinase^a^	HV10_00603	–
	*pgl*	6-phosphogluconolactonase^a^	HV10_00604	–
	zwf	Glucose-6-phosphate dehydrogenase^a^	HV10_00605	–
	*edd*	Phosphogluconate dehydratase^a^	HV10_00606	–
	*dgoA*	Ketohydroxglutarate aldolase^a^	HV10_00607	–
Stress response	*-*	D-beta-hydroxybutyrate permease	HV10_00717	Up/1.61
	*-*	D-beta-hydroxybutyrate dehydrogenase	HV10_00718	Up/1.86
	*atoE*	Short-chain fatty acids transporter	HV10_00719	Up/l1.73
	*scoB*	Succinyl-coA: 3-ketoacid coenzyme A tranferase	HV10_00720	Up/1.48
	*scoA*	Succinyl-coA: 3-ketoacid coenzyme A tranferase	HV10_00721	Up/1.45
	-	Putative acetyl-coA acyltransferase	HV10_00722	Up/logFC 1.10
	*ald2*	Alanine dehydrogenase	HV10_00723	–
Sulfur assimilation	*cysD*	Sulfate adenyltransferase small subunit	HV10_01024	Up/logFC 1.68
	*cysN*	Sulfate adenyltransferase subunit	HV10_01025	–
	*-*	SLC13 family permease	HV10_01026	–
	*cysC*	Adenylyl-sulfate kinase	HV10_01027	–
Pseudaminic acid biosynthesis	*pseI*	pseudaminic acid synthase	HV10_095	–
	*pseA*	N-acetyl sugar amidotransferase	HV10_096	–
	*pseH*	UDP-4-amino-4%2C6-dideoxy-N-acetyl-beta-L-altrosamine N-acetyltransferase	HV10_099	–
	*pseG*	UDP-6-deoxy-AltdiNAc hydrolase (PseG, third step of pseudaminic acid biosynthesis)	HV10_100	–
	*pseF*	Pseudaminic acid cytidylyltransferase	HV10_101	–
	*pseC*	C4 aminotransferase specific for PseB product (PseC, second step of pseudaminic acid biosynthesis)	HV10_108	–
	*pseB*	UDP-N-acetylglucosamine 4,6-dehydratase (inverting)	HV10_109	Down/logFC −1.34
Bile Resistance, antibiotic resistance, colonization	*cmeA*	Efflux RND transporter periplasmic adaptor subunit	HV10_01506	–
	*cmeB*	RND transporter permease subunit	HV10_01505	–
	*cmeC*	TolC family protein/outer membrane protein	HV10_01504	–
	*cmeR*	TetR/AcrR family transcriptional regulator	HV10_01507	Up/logFC 2.35
	*acrB*	acrB/acrD/acrF family protein	HV10_00366	–
	*acrA*	RND transporter periplasmic adaptor subunit	HV10_00367	–
	*toIC*	TolC family protein/outer membrane protein	HV10_00368	–
Oligopeptide transporter^a^	*oppA*	Peptide ABC transporter substrate binding protein^a^	HV10_00013	–
	*oppB*	ABC transporter permease^a^	HV10_00014	–
	*oppC*	ABC transporter permease^a^	HV10_00015	Down/logFC −1.35
	*oppC−3‘ fragment*	ABC transporter permease^a^	HV10_00016	–
	*oppD*	ABC transporter ATP-binding protein^a^	HV10_00017	Down/logFC −1.67
	*oppF–fragment*	ABC transporter ATP-binding protein^a^	HV10_00018	–
	*oppF–fragment*	ABC transporter ATP-binding protein^a^	HV10_00019	Down/logFC −1.84
	*papP*	Amino acid ABC transporter permease	HV10_00052	Up/logFC 1.10
	*CJ14980A_0432*	Amino acid ABC transporter permease	HV10_00053	Up/logFC 1.41
	*papQ*	Amino acid ABC transporter ATP-binding protein	HV10_00054	Up/logFC 1.24
Hydrogenase (electron donor–anaerobic motility)	*hydA*	Ni/Fe hydrogenase small subunit	HV10_00135	Up/logFC 1.08
	*hydB*	Ni/Fe hydrogenase large subunit	HV10_00136	Up/logFC 0.97
	*hydC*	Ni/Fe hydrogenase b-type cytochrome subunit	HV10_00137	Up/logFC 1.03
	*hydD*	Ni/Fe hydrogenase expression/formation protein	HV10_00138	Up/logFC 1.53
	*hypA*	Ni metallochaperone	HV10_00708	–
	*hypE*	Hydrogenase expression/formation protein	HV10_00709	Up/logFC 1.23
	*hypD*	Hydrogenase formation protein	HV10_00710	Up/logFC 1.21
	*hypC*	Hydrogenase formation protein	HV10_00711	Up/logFC 1.94
	*hypB*	Hydrogenase formation protein	HV10_00712	Up/logFC 1.51
	*hypF*	carbamoyltransferase	HV10_00713	–
Oxidative phosphorylation	-	NADH dehydrogenase (EC 1.6.99.3)	HV10_00727	Down/logFC−3.08
	-	NADH dehydrogenase	HV10_00728	Down/logFC −3.17
	*-*	NADH ubiquinone oxidoreductase chain A (EC 1.6.5.3)	HV10_1399	–
	*-*	NADH-ubiquinone oxidoreductase chain B (EC 1.6.5.3)	HV10_1400	–
	*-*	NADH-ubiquinone oxidoreductase chain C (EC 1.6.5.3)	HV10_1401	–
	*-*	NADH-ubiquinone oxidoreductase chain D (EC 1.6.5.3)	HV10_1402	–
	*-*	NADH-ubiquinone oxidoreductase chain E (EC 1.6.5.3)	HV10_1403	–
	*-*	NADH-ubiquinone oxidoreductase chain G (EC 1.6.5.3)	HV10_1405	–
	*-*	NADH-ubiquinone oxidoreductase chain H (EC 1.6.5.3)	HV10_1406	Up/logFC 1.28
	*-*	NADH-ubiquinone oxidoreductase chain I (EC 1.6.5.3)	HV10_1407	Up/logFC 1.63
	*-*	NADH-ubiquinone oxidoreductase chain J (EC 1.6.5.3)	HV10_1408	Up/logFC 1.85
	*-*	NADH-ubiquinone oxidoreductase chain K (EC 1.6.5.3)	HV10_1409	Up/logFC 1.88
	*-*	NADH-ubiquinone oxidoreductase chain L (EC 1.6.5.3)	HV10_1410	Up/logFC 1.34
	*-*	NADH-ubiquinone oxidoreductase chain M (EC 1.6.5.3)	HV10_1411	Up/logFC 1.48
	*-*	NADH-ubiquinone oxidoreductase chain N (EC 1.6.5.3)	HV10_1412	Up/logFC 1.30
	*-*	Ubiquinol-cytochrome C reductase iron-sulfur subunit (EC 1.10.2.2)	HV10_212	Up/logFC 1.55
	*-*	Ubiquinol–cytochrome c reductase, cytochrome B subunit (EC 1.10.2.2)	HV10_213	Up/logFC 1.94
	*-*	Ubiquinol cytochrome C oxidoreductase, cytochrome C1 subunit	HV10_214	Up/logFC 2.07
Phosphate metabolism	*pstB*	Phosphate transport ATP-binding protein PstB (TC 3.A.1.7.1)	HV10_00729	–
	*pstA*	Phosphate transport system permease protein PstA (TC 3.A.1.7.1)	HV10_00730	–
	*pstC*	Phosphate transport system permease protein PstC (TC 3.A.1.7.1)	HV10_00731	Up/logFC 2.04
	*pstS*	Phosphate ABC transporter, periplasmic phosphate-binding protein PstS (TC 3.A.1.7.1)	HV10_00732	Up/logFC 2.01
Formate dehydrogenase (electron donor–anaerobic motility)	*fdhA 5' fragment*	Formate dehydrogenase	HV10_00818	–
	*fdhA*	Formate dehydrogenase subunit alpha	HV10_00819	–
	*fdhB*	Formate dehydrogenase subunit beta	HV10_00820	–
	*fdhC*	Formate dehydrogenase subunit gamma	HV10_00821	–
	*fdhD*	sulfurtransferase	HV10_00822	Down/logFC −2.09
Capsule locus (CAP)	*kpsS*	Capsule biosynthesis protein	HV10_00976	Down/logFC −1.05
	*kpsC*	Capsule biosynthesis protein	HV10_00977	–
	-	36 CDS–capsule related genes and other	HV10_00978 to HV10_01013	9 Down, 27 –
	*tagG*	Capsule biosynthesis protein	HV10_01014	–
	*kpsT*	ABC transporter ATP-binding protein	HV10_01015	–
	*kpsE*	Capsule biosynthesis protein	HV10_01016	Down/logFC −1.33
	*kpsD*	Sugar ABC transporter substrate binding protein	HV10_01017	–
	*kpsF*	Sugar phosphate isomerase	HV10_01018	–
	*-*	Sugar transferase	HV10_01019	–
	*-*	Capsule biosynthesis protein	HV10_01020	–
	*-*	Hypothetical protein	HV10_01021	–
	*-*	Glycosyltransferase family A protein	HV10_01022	–
	*-*	Polysaccharide biosynthesis protein^c^	HV10_00267	–
	*galE*	UDP-glucose 4-epimerase^3^	HV10_00268	–
	*-*	ABC transporter ATP binding protein^c^	HV10_00269	Up/logFC 1.12
	*-*	Glycosyltransferase family 4 protein^c^	HV10_00270	–
	*-*	Glycosyltransferase family 2 protein^c^	HV10_00271	Up/logFC 1.04
	*-*	Glycosyltransferase^c^	HV10_00272	–
	*-*	Peptide binding protein^c^	HV10_00273	–
	*-*	Glycosyltransferase family 1 protein^c^	HV10_00274	–
	*-*	Sugar transferase^c^	HV10_00275	–
	*-*	Acetyltransferase^c^	HV10_00276	–
	*degT*	Aminotransferase^c^	HV10_00277	–
	*FlaA1*	Polysaccharide polyermase^c^	HV10_00278	Up/logFC 1.01
Lipooligosaccharide locus (LOS) Rearrangement and recombination events (seems common place in literature) Outer core glycosyltransferases between WaaF and WaaC Ganglioside mimics (NeuABC) rearranged outside of locus.	*ccds*	Biofunctional heptose 7-phosphate kinase	HV10_00242	Up/logFC 1.59
	*gmh*	Phosphoheptose isomerase	HV10_00243	–
	*neuA*	Actylneuraminate cytidylyltransferase	HV10_00244	–
	*neuC*	UDP-N-acetylglucosamine	HV10_00245	Up/logFC 1.10
	*neuB*	N-acetlyneuaminate synthase	HV10_00246	Up/logFC 1.18
	*cst-1*	Alpha-2,3-sialytransferase	HV10_00247	–
	*waaF*	Lipopolysaccharide heptosyltransferase II	HV10_00248	–
	*waaV*	glucosyltransferase	HV10_00249	Down/logFC −1.36
	*-*	Glycosyltransferase family 4 protein	HV10_00250	Down/logFC −1.23
	*-*	Glycosyltransferase family 4 protein	HV10_00251	Down/logFC −6.69
	*-*	Glycosyltransferase family 2 protein	HV10_00252	–
	*-*	Glycosyltransferase family 2 protein	HV10_00253	–
	*-*	Glycosyltransferase family A protein	HV10_00254	–
	*cgtA fragment*	Beta-1,4-N-acetylgalactosaminyltransferase	HV10_00255	–
	*fragment*	Glycosyltransferase family 2 protein^b^	HV10_00256	–
	*-*	Glycosyltransferase family 8 protein^b^	HV10_00257	–
	*-*	Glycosyltransferase family 4 protein^b^	HV10_00258	–
	*-*	Glycosyltransferase family 2 protein^b^	HV10_00259	–
	*-*	Glycosyltransferase family 2 protein^b^	HV10_00260	Down/logFC −2.04
	*-*	Glycosyltransferase family 2 protein^b^	HV10_00261	–
	*fragment*	Glycosyltransferase family A protein^b^	HV10_00262	–
	*cgtA fragment*	Beta-1,4-N-acetlygalactosamintyltransferase	HV10_00263	–
	*-*	Glycosyltransferase family 2 protein	HV10_00264	Down/logFC −1.08
	*waaM*	Lauroyl acyltransferase	HV10_00265	–
	*waaC*	Lipopolysaccharide heptosyltransferase I	HV10_00266	Up/logFC 1.04
Subtype II CRISPR	*-*	Type II CRISPR RNA-guide endonuclease cas9–partal	HV10_01290	–
	*-*	Type II CRISPR RNA-guide endonuclease cas9	HV10_01291	–
	*-*	Type II CRISPR RNA-guide endonuclease cas1	HV10_01292	–
	*-*	CRISPR-associated endonuclease cas2	HV10_01293	–
	*-*	Multiple cds of small fragments/all hypothetical		
	*-*	CRISPR-associated endonuclease cas2	HV10_01316	–

* Blastp based on amino acid identity.

a Integration of operon in between 16S rDNA and 23S rDNA.—might be acquired through horizontal gene transfer. Two of the three 16 rDNA operons have gene integrations.

b Potential rearrangement/insertion.

c *Roles in both CAP and LOS—located downstream of LOS locus*.

Various methods of genome comparison have been used to investigate coding sequences that are unique to *C. hepaticus*. The strongest associations with high specificity and selectivity to *C. hepaticus* were genes with predicted roles in chemotaxis, capsule and lipooligosaccharide synthesis and metabolism. Four chemotaxis proteins with < 88% homology to known chemotaxis proteins were characterized, which could play a role in the movement of *C. hepaticus* from the gastrointestinal tract to the liver (Table 2). Significant variation was also characterized in the lipooligosaccharide locus (LOS), a region of the *Campylobacter* chromosome known to undergo rearrangements and recombination events (Parker et al., 2005; Revez and Hänninen, 2012). Two points of interest in this locus were exclusive to *C. hepaticus*. Firstly the ganglioside mimics (NeuABC) are rearranged outside of the locus as normally seen in *C. jejuni* (no longer located between the Waac/WaaM to WaaV/WaaF). Secondly, there was an apparent ~6.6 kb insertion of seven CDS into the cst-II gene, all with functions predicted as various glycosyltransferases. This insertion in the middle of the locus resulted in the truncation of *cgtA* (Table 2). Roughly 2 kb of the inserted sequence is unique to *C. hepaticus*, with the remaining 4.6 kb showing high sequence divergence to *C. jejuni* isolates.

A glucose utilization operon was found to be associated with SLD and is discussed in detail in a later section. All C*. hepaticus* isolates encode a region of CRISPR-cas genes (type II-cas9 CRISPR), however a CRISPR array (section of repeats and spacers) was only found (CRISPR-finder) in two isolates from the divergent Australian Cluster 2 (three direct repeats and 2 spacers, isolates 19L and 54L). The remaining 22 isolates did not encode a complete CRISPR array, just the cas genes (*cas9, cas1* and a fragmented *cas2*). A region of 13 kb is inserted within the two *cas2* CDS of the 22 remaining isolates; ~7kb is unique to *C. hepaticus*. The GC content of this region is similar to that of the rest of the chromosome (28.03%), with most genes (11 CDS) having unknown functions. Within this region, three CDS encoding for luxA repressor, XRE family transcriptional regulation and type II toxin-antitoxin system mRNA interferase are present. A screen for prophage using PHASTER did not identify any complete prophage integrations within any of the genomes.

### Horizontally Acquired-Elements: Plasmids

Plasmids are present in five out of fourteen *C. hepaticus* Australian isolates (Table 3). Using ABRicate to screen for antibiotic resistance genes, a single antibiotic resistance gene, *tetO*, was found in eight isolates (5 from Australia and 3 from the UK), which correlated directly to the presence of plasmid elements. Distinct plasmids were found based on the country of origin of the isolates. UK isolates contained a plasmid highly homologous to the previously characterized *C. coli* plasmid pCC31 (99% coverage and identity), while the Australian isolates contain plasmids homologous to the *C. jejuni* pCJDM210L plasmid (93% coverage and 99% identity). This plasmid harbored a type IV secretion system along with a tetracycline-resistant gene. Five of the Australian isolates within this study (27L, 84B, Ace1, Ace8659, and AceM3a) carry the plasmid and it accounts for roughly half of the gene content within the accessory genome of these isolates. As short-read sequence data was used it was not possible to assemble the plasmid in its entirety. At least three contigs from each of these genomes were highly conserved and carried plasmid elements.

**Table 3:** Plasmid contents of *C. hepaticus* Australian and UK isolates.

**Strain**	**ACE1**	**ACE8659**	**ACEM3A**	**84B**	**27L**	**S11-010**	**S12-002**	**S12-0322**
Country	AUS	AUS	AUS	AUS	AUS	UK	UK	UK
Plasmid closest hit^*^	a	a	a	a	a	b	b	b
Contigs	3	3	3	3	3	1	1	1
Size (kbp)	~44.4	~45.3	~44.9	~44.9	~44.9	~44.8	~44.9	~44.9
GC %	28.4	28.3	28.3	28.3	28.3	29.7	29.1	29.6
*tetO*	1	1	1	1	1	1	1	1

* *a: pCJDM210L (C. jejuni); b: pCC31 (C. coli)*.

### Horizontally Acquired-Elements: Insertions in Ribosomal RNA Operons

**Figure 3:**
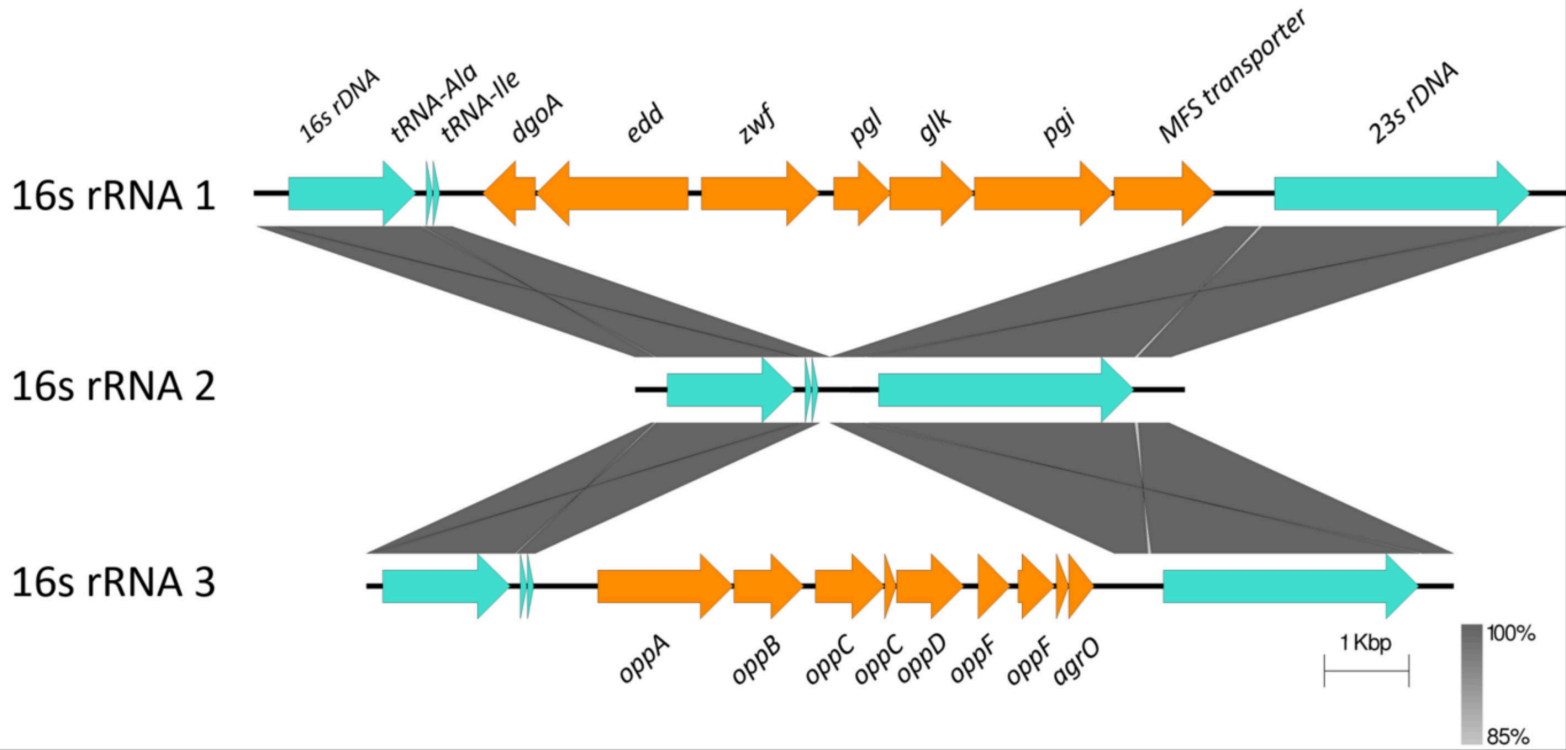
Sequence alignment of the three ribosomal RNA gene operons from *C. hepaticus* HV10. The conserved 16S, 23S, and tRNA are colored in pale blue, while the insertion regions are colored orange. The blastn and sequence alignment figure were produced in Easyfig v2.2.2 (Sullivan et al., 2011).

*C. hepaticus* encodes three ribosomal RNA operons, however two have been disrupted by the insertion of multiple CDS between the 16S and the 23S genes. A glucose utilization operon and an oligopeptide transporter operon were located within ribosomal RNA operons (Figure 3). Analysis of the insertions showed that the glucose utilization and oligopeptide transporter regions have GC content of 28.17 and 27.56% respectively, which is similar to the average GC content of the HV10 genome, 28.2%.

### Confirmation of Glucose Utilization Ability in *C. hepaticus*

*C. hepaticus* cultures incubated for 24 h in IS media containing L-cysteine and D-glucose-6-phosphate, or L-cysteine and D-glucose showed color development (due to TTC) and therefore demonstrated utilization of the substrates, whereas C*. jejuni* and *C. coli* cultures did not. There was no color development in *C. hepaticus* cultures incubated in IS plus L-cysteine only or IS plus D-glucose or D-glucose-6–phosphate. This was due to a lack of carbon source and nitrogen source, respectively. The color change was observed in all cultures grown in Brucella broth, demonstrating the viability of the inoculated cultures.

### Gene Expression in *C. hepaticus* Recovered From Bile

**Figure 4:**
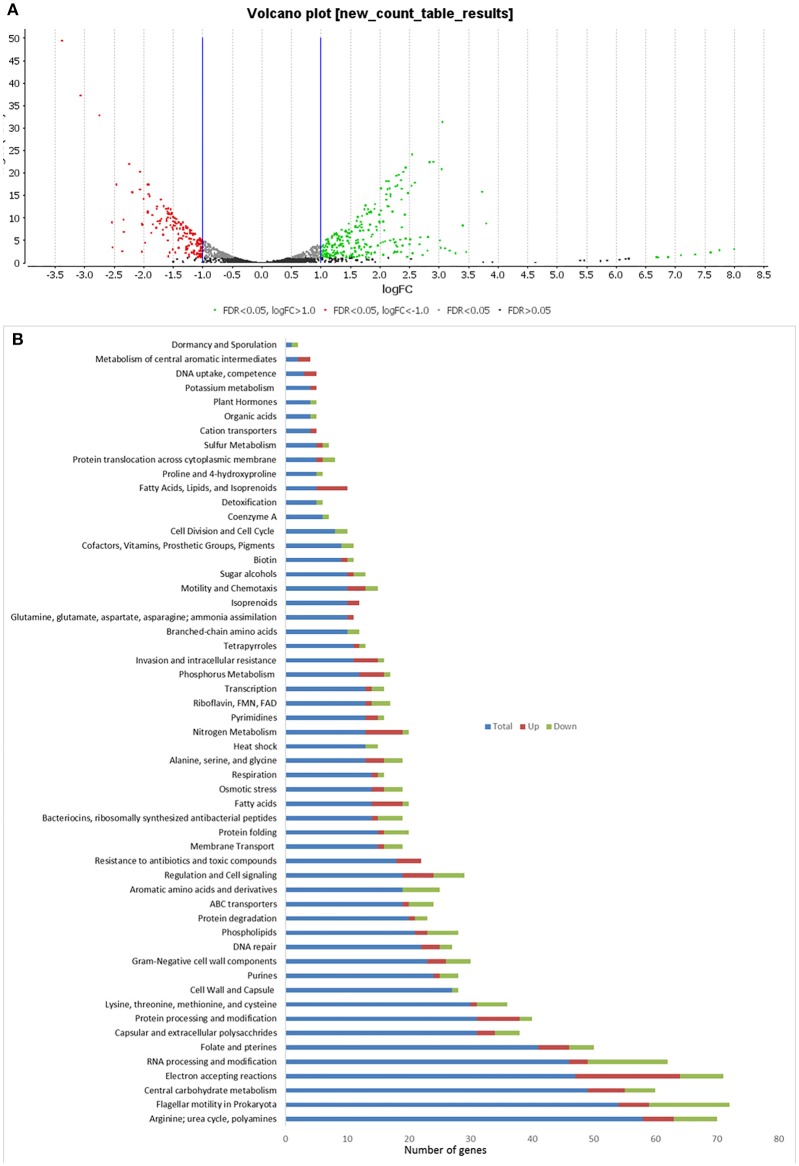
Comparison of differentially expressed genes identified between *in vitro* and *in vivo* conditions. **(A)** Volcano plots analysis of differentially expressed genes DEGs. The green dots represent DEGs up regulated in bile samples, the red dots represent DEGs down-regulated in bile samples, and the black/gray dots represent no DEGs. **(B)** Sub-categories of DEGs were as defined by the SEED viewer from the RAST annotations. Total: number of CDSs assigned to each subcategory, Up: DEGs up regulated while *C. hepaticus* in bile samples compared to *in vitro* samples.

**Figure 5:**
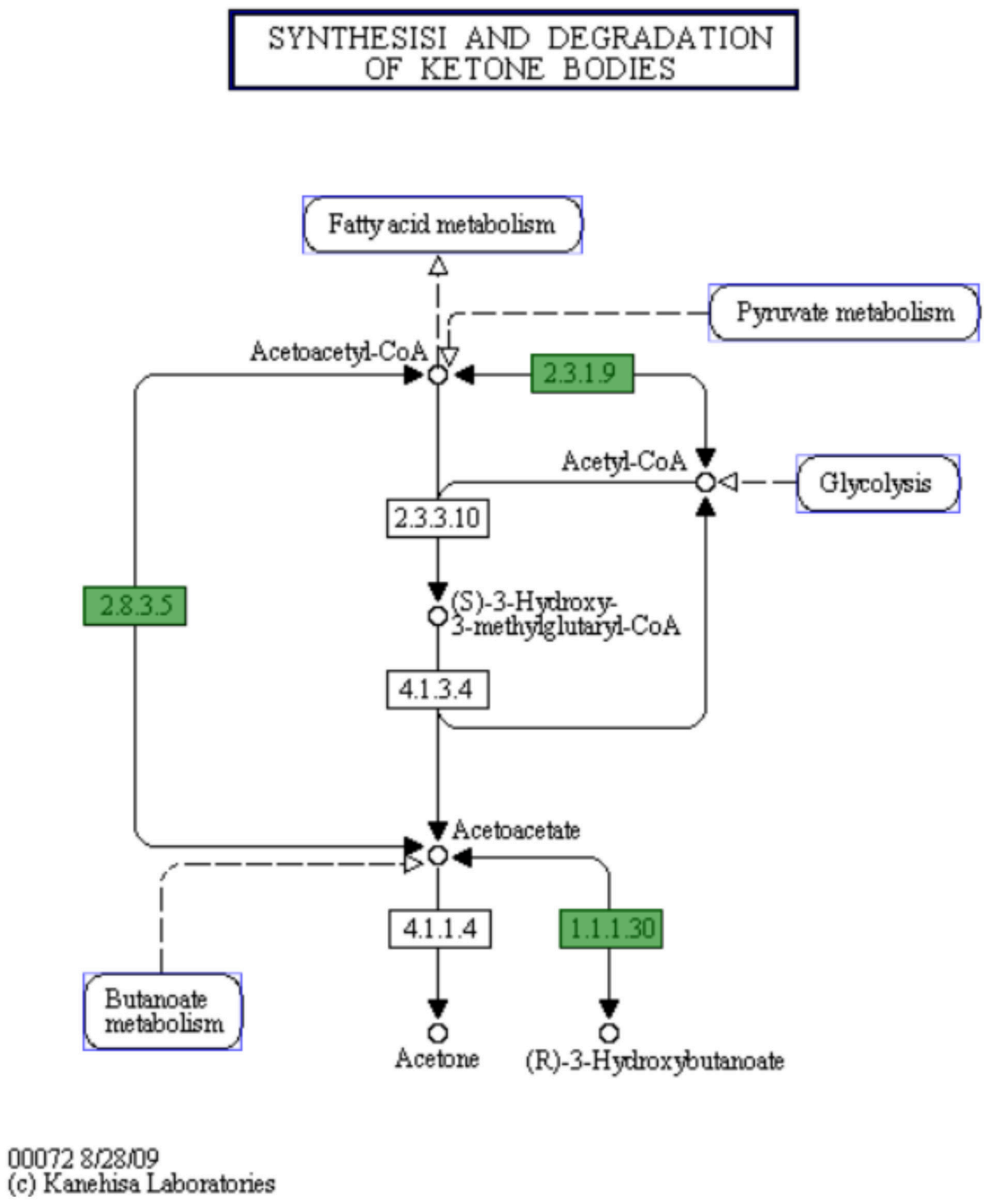
The putative biosynthetic pathway for PHB metabolites in *C. hepaticus* (KEGG map generated from the SEED Viewer*)*. Genes in green boxes are present in *C. hepaticus*. EC 1.1.1.30: D-beta-hydroxybutyrate dehydrogenase. EC 2.3.1.9: Acetyl-CoA acetyltransferase. EC 2.8.3.5: Succinyl-CoA:3-ketoacid-coenzyme A transferase.

In *C. hepaticus* recovered from the gall bladder of SLD experimentally infected birds, 410 genes were differentially expressed (False Discovery Rate (FDR) < 0.05) when compared to *in vitro* grown bacteria. There were 164 up-regulated genes ((log2-fold-changes) > 1.0) *in vivo* and 246 down-regulated genes (logFC < −1.0). Functional gene categorization assessed using the SEED Viewer, showed that the 410 differentially expressed genes belonged to 56 subcategories (Figure 4). Notably, all genes associated with polyhydroxybutyrate (PHB) metabolism (Figure 5) were up-regulated (EC 1.1.1.30: D-beta-hydroxybutyrate, EC 2.3.1.9: Acetyl-CoA acetyltransferase, EC 2.8.3.5: Succinyl-CoA:3-ketoacid-coenzyme A transferase, and genes encoding D-beta-hydroxybutyrate permease, short chain fatty acids transporter and 3-ketoacyl-CoA thiolase). These genes may play a role in stress response in *C. hepaticus* and are putative virulence factors (Table 2).

The gene clusters encoding Ni-Fe-hydrogenase were up-regulated in the cells recovered from bile (Table 2). Six of out eight genes associated with nitrate and nitrite ammonification (nitrogen metabolism system) were also up-regulated and only one was down-regulated in bile samples (Figure 4B). Transcripts from the phosphate transport system of *pstS* and *pstC* were increased in abundance *in vivo* compared to in *vitro* (Table 2). RNA-Seq identified increased abundance of many transcripts associated with copper homeostasis and up-regulation of pathogenesis-associated glutamine ABC transporters, *papP* and *papQ* (Table 2). The *neuB* (N-acetylneuraminate synthase) and *neuC* (UDP-N-acetylglucosamine) genes, necessary for sialic acid synthesis, were both up-regulated in the bile-derived bacteria. Thirteen genes associated with flagella motility were down-regulated, and only five were up-regulated. Increased expression of flagella associated genes included genes encoding flagella motor protein (MotA) and flagella switch motor protein (FliN). Down-regulated genes included a putative lipoprotein required for motility, a motility integral membrane protein and flagella-associated genes including *flgB, flgD, flgF, flgG, flgH*, and *flgI*. In addition, many genes in the aromatic amino acids and derivatives category were down-regulated (Figure 4B), including genes involved in common pathways for synthesis of aromatic compounds, tryptophan synthesis, and chorismate synthesis (intermediate for synthesis of tryptophan) and none of the genes in this category were up-regulated. Similarly, genes associated with the production of methionine (lysine, threonine, methionine, and cysteine subcategory, Figure 4B) were down-regulated. RNA-Seq identified decreased abundance of transcripts associated with tRNA processing, RNA methylation, and RNA pseudouridine syntheses. On the other hand, there was variation in the expression of genes involved in the oxidative phosphorylation pathway, as genes encoding the enzyme NADH dehydrogenase (EC 1.6.99.3) were down-regulated, while many genes encoding enzymes NADH ubiquinone oxidoreductase (EC 1.6.5.3) and ubiquinol-cytochrome C reductase (EC 1.10.2.2) were up-regulated (Table 2).

## Discussion

*Campylobacter hepaticus* core genome phylogeny showed five phylogroups, two from Australian isolates and three from UK isolates. Interestingly, the main Australian phylogroup includes all the isolates from southern Australia (Victoria, South Australia, New South Waves) while the two Queensland isolates (northern Australia) formed a separate phylogroup. This indicates that *C. hepaticus* clonal populations are geographically confined.

The comparison of *C. hepaticus* genomes with those of representative isolates of *C. jejuni* and *C. coli* indicated that there are barriers to gene flow among these related populations, even though these species are known to be common colonizers of commercial poultry and are naturally transformable (Vegge et al., 2012). This suggests a mechanistic barrier to homologous recombination or an adaptive selection against hybrid genotypes, possibly influenced by the reduced genome size of *C. hepaticus* (0.2–0.4 Mbp reduction), reduced metabolic capabilities, and a reduced GC content (2–3.5% lower than *C. jejuni* and *C. coli*). The genetic divergence of *C. hepaticus* from other *Campylobacter* spp. is likely due to its adaptation to colonize and infect the bile and liver in chickens.

In *C. jejuni*, the cytolethal distending toxin (CdtA, B, C) has been recognized as a major virulence factor and is believed to induce host cell apoptosis (Dasti et al., 2010). However, the Cdt is not encoded by *C. hepaticus* and the genomic analysis has not identified any other candidate toxin genes. *C. hepaticus* has a large number of genes associated with chemotaxis (11 genes), motility (47 genes), and adherence/antigen presentation (45 genes); genes similar to many that have been shown to be required for the colonization and infection of other bacteria. In addition, *C. hepaticus* encodes *Campylobacter* invasion antigens (*CiaB*), presumably secreted from the flagella export apparatus. In the case of *C. jejuni*, this protein has been demonstrated to be delivered to the host cell cytoplasm, which stimulates host cell signaling and prompts bacterial internalization (Konkel et al., 2004). The CiaB antigen plays a major role in the invasion of chicken epithelial cells. Mutants which lack the *ciaB* gene were shown to have reduced virulence (Ziprin et al., 2001; Biswas et al., 2007). *In vitro* assays using chicken epithelial cells have demonstrated that *C. hepaticus* is invasive, probably more so than *C. jejuni* (Van et al., 2017a). *C. hepaticus* also encodes a set of genes involved in pseudaminic acid biosynthesis (Pse). The structural flagellin proteins of *Helicobacter pylori* and *Campylobacter jejuni* are glycosylated with Pse and this glycosylation is essential for flagella filament assembly and consequent motility, therefore Pse is considered to be a key virulence factor (Ménard et al., 2014).

To elucidate the genetic potential of *C. hepaticus* to cause SLD various genome comparison tools were used to screen each gene in the *C. hepaticus* pan genome for association to SLD. An association study was used to search for genes or markers associated with SLD, and genes with predicted roles in chemotaxis, capsule and lipooligosaccharide synthesis and metabolism were identified (Table 2). Four chemotaxis proteins with low identity to known chemotaxis proteins (< 88%) were identified and two of these genes were up-regulated *in vivo*, in the *C. hepaticus* recovered from bile. These genes could play a role in the movement of *C. hepaticus* from the gastrointestinal tract to the liver and bile and are priority gene targets for further study.

A screen for prophage insertions into the genome using PHASTER failed to identify any prophage integrations within the genomes. The lack of a CRISPR spacer array suggests the CRISPR region is not actively used as an immune system for *C. hepaticus*. Type II cas9 systems in *C. jejuni* and *Neisseria meningititis* are required for the ability to invade, attach to and replicate within epithelial cells (Sampson and Weiss, 2013), although mechanisms are currently unknown. Cas9 has been correlated with strains producing sialylated lipooligosaccharide structures in the outer envelope (Sampson and Weiss, 2013). However, there is a unique ~7kb insertion within the two *cas9* CDS, with found exclusively with *C. hepaticus* isolates. This insertion encodes many genes with unknown function including three CDS encoding for luxA repressor, XRE family transcriptional regulation and type II toxin-antitoxin system mRNA interferase. This indicates this region may play a regulatory role in *C. hepaticus*, possible affecting virulence.

Although the *C. hepaticus* genomes do not appear to be highly influenced by horizontal gene transfer and acquisition of genetic material, there are three regions, two chromosomal and one plasmid, associated with lateral gene transfer events. Glucose utilization and oligopeptide transporter operons were located within two of the three ribosomal RNA operons (Figure 3). It is unusual to have a large operon inserted between the 16S and 23S rRNA genes, although strains *C. coli* CHW470 and *C. jejuni* subsp. *Doylei* 269.97 were found to have glucose utilization operons inserted between 16S rRNA and 23S rRNA genes (Vorwerk et al., 2015). The region which lays between the 16S rRNA and 23S rRNA genes, called the Internal Transcribed Spacer (ITS) region, of other *Campylobacter* species were highly variable in % GC content and length, with an average size of 880 bp, and the longest was 1,646 bp in *C. hominis* ATCC BAA 381 (Man et al., 2010).

Typically, *Campylobacter* species are characterized as non-glycolytic bacteria. *C. hepaticus* contain many more genes in carbohydrate utilization pathways than *C. jejuni* and this may help *C. hepaticus* to survive in the carbohydrate-rich environment of the chicken liver (Petrovska et al., 2017). The presence of the glucose utilization operon enables the metabolism of glucose through the glycolytic (Entner-Doudoroff, ED) pathway and has previously been found in other bacteria such as *Helicobacter* (Hofreuter, 2014). Most *C. jejuni* and *C. coli* genomes do not have genes encoding glucokinase (EC.2.7.1.2), glucose-6-phosphate dehydrogenase (EC 1.1.1.49) and 6-phosphogluconolactonase (EC 3.1.1.31) and are therefore mostly ED-negative. Vegge *et al*. found that only 1.7% of >6,000 genomes of *C. coli* and *C. jejuni* encoded a complete ED pathway (Vegge et al., 2016). From the fully closed and finished genome of *C. hepaticus* HV10, three rRNA were identified, two of which were disrupted by a glucose utilization oligopeptide transporter operons. All the genes in these two operons are present in all the other *C. hepaticus* isolates. The bioinformatics prediction of D-glucose utilization by *C. hepaticus* was experimentally confirmed and both *C. jejuni* NCTC 11828 and *C. coli* NCTC 11366 were shown to be unable to utilize D-glucose. Vorwekr et al. demonstrated the ED pathway of glucose-catabolising *C. coli* strains could be acquired by non-glycolytic *C. coli* isolates through natural transformation, showing that the ED pathway genes could be transferred by horizontal gene transfer (Vorwerk et al., 2015). In *C. hepaticus*, the GC content of these two regions (27.56% and 28.17%) are similar to the average GC content of the HV10 genome (28.2%), suggesting that these have been present within the genome for an extended period of time, or have been obtained from a close relative. In contrast, the GC content of ED pathway genes in *C. coli* CHW470 are 34.7–36.5% while the GC content of the isolate is 31.1%. These loci are present in all the *C. hepaticus* isolates and therefore it is suggested that these loci might provide a selective advantage. Carbon source utilization is characteristic of growth of intercellular gastrointestinal pathogens such as *Listeria monocytogenes* and *Salmonella* Typhimurium (Dandekar et al., 2012; Fuchs et al., 2012), therefore the high level of conservation of this locus in *C. hepaticus* may provide a new pathway for pathogenesis of SLD.

The presence of two distinct tetracycline resistant plasmids that appear to originate from two distinct species suggest that other *Campylobacter* species may act as a genetic reservoir for *C. hepaticus* and vice versa, which is likely due to the presence of the type II secretions systems (transformation locus) present in *C. hepaticus*. However, as two different plasmids are present in the *C. hepaticus* sampled here and only in 1/3 isolates (absent from HV10), this suggests that the genes encoded on plasmids do not play a role in SLD development. The *C. coli* pCC31 plasmid has been shown to be conjugative (Batchelor et al., 2004); therefore, the closely related plasmid found in some *C. hepaticus* isolates may also be transferable. This is concerning as this antibiotic resistance plasmid could be disseminate to other bacteria. This should be taken as an early warning sign that alternative treatments, other than antibiotic treatment, are needed for the control of SLD.

PHB is produced by microorganisms in responses to physiologically stressed conditions, especially when nutrients are limited (Ackermann et al., 1995; Batista et al., 2018). In *C. hepaticus*, PHB might be produced by the condensation of acetyl-CoA to acetoacetyl-CoA and is later converted to acetoacetate, and acetoacetate is then reduced by NADH to *R*-3-hydroxybutyrate where D-beta-hydroxybutyrate dehydrogenase enzyme catalyzes the reaction (Figure 5). In contrast, *C. jejuni* and *C. coli* lack this pathway. A Biolog Phenotype Microarray confirmed the metabolic activity of D-beta-hydroxybutyrate dehydrogenase as the color change was observed in the wells with acetoacetate substrate and *C. hepaticus* added but not in wells with *C. jejuni* (data not shown). All genes associated with PHB metabolism were up-regulated. PHB is accumulated by bacteria as a carbon and energy storage when carbon sources are freely available but limited for other nutrients (Ratcliff et al., 2008; Reusch, 2013).

Sialic acid has been demonstrated to shield pathogens from host immune responses by interacting with the sialic acid-binding proteins of the host. For example, Group B *Streptococcus* (GBS) can evade host responses and proliferate in blood due to capsular polysaccharide displaying sialic acid residues (Chang et al., 2014; Lewis et al., 2016). *C. hepaticus* harbors a sialic acid biosynthetic gene locus (UDP-GlcNAc converts to ManNAc, then converts to Neu5 Ac, followed by CMP-Neu5Ac, with the action of NeuC (EC 5.1.3.14), NeuB (EC 2.5.1.56) and NeuA (N-Acetylneuraminate cytidylyltransferase, EC 2.7.7.43, respectively). The RNA-Seq analysis showed up-regulation of *neuB* and *neuC* in the bile environment, suggesting the sialic acid biosynthetic genes may encode a host immune response avoidance mechanism.

The gene clusters encoding Ni-Fe-hydrogenase were up-regulated in the *in vivo* cells recovered from bile. Hydrogenases catalyze the reversible reaction: 2H^+^ + 2e^−^ ⇔ H_2_ and they play an important role in dealing with fluctuations in energy and oxygen supply (Vignais et al., 2001). In *H. pylori*, H_2_ produced by the gastric microbiota serves as a respiratory substrate which substantially enhances its ability to colonize the stomach (Olson and Maier, 2002). Similarly, the hydrogenase may function as a virulence factor in *C. hepaticus*.

The pathogenesis-associated glutamine ABC transporter genes, *papP* and *papQ* were up-regulated in the *in vivo* cells. This was expected as glutamine is the only amino acid that contains an additional nitrogen molecule and the liver is the major site of nitrogen metabolism (Haüssinger, 1990). PaqP and PaqQ have been demonstrated to play a role in bacterial stress tolerance and pathogenesis of *C. jejuni* (Lin et al., 2009). Genes encoding products involved in copper homeostasis were up regulated *in vivo*. This may explain the survival ability of *C. hepaticus* in the high copper environment of bile.

Depending on cell growth and metabolism needed to adapt to a new environment, proteins are produced and mRNAs, tRNAs, and rRNAs are all orchestrated to accomplish their roles (Arraiano et al., 2010). It is no surprise that many genes involved in RNA metabolism and genes associated with synthesis of amino acids in *C. hepaticus* in bile samples were down-regulated, as it appears that the bacterium was in a somewhat quiescent, resting stage in bile.

Up-regulation of a phosphate transport system *pstSCAB* was observed in *C. jejuni in vivo* (caecum) compared to *in vitro* conditions (Taveirne et al., 2013). In our study, up regulation of *pstS* and *pstC* in bile samples compared to *in vitro* samples was also observed, suggesting that the bile environment is limited in phosphate. A study by Stintzi *et al*. found that the expression of genes encoding NADH dehydrogenase and succinate dehydrogenase were decreased in rabbit intestines. This is consistent with the oxygen-limited environment of the intestine (Stintzi et al., 2005). However, the situation seems to be more complex in bile. In the oxidative phosphorylation pathway, there was decreased expression of the genes encoding enzyme NADH dehydrogenase, while genes in this pathway encoding enzymes such as NADH ubiquinone oxidoreductase and ubiquinol-cytochrome C reductase were up-regulated.

*Campylobacter jejuni* can use a wide range of alternative electron acceptors to oxygen, including fumarate, nitrate, nitrite, and *N*- or *S*-oxides, under oxygen-restricted conditions *in vitro* (Sellars et al., 2002). *C. hepaticus* HV10 encodes a number of reductases including fumarate reductase and a nitrate reductase of the periplasmic Nap type. Our study showed up-regulation of all genes encoding nitrate reductase, suggesting a *C. hepaticus* response to the oxygen-limited environment found in bile.

Bacterial flagellum is a complex apparatus assembled of more than 20 different proteins (Haiko and Westerlund-Wikström, 2013). Flagella can play an essential role in colonization of many bacteria by facilitating bacterial motility. They also have adhesive and invasive properties and act as potential virulence factors. Many genes involved in flagella and chemotaxis were found to be down-regulated in bacteria recovered from bile. This indicates there may be no requirement for facilitating bacterial motility once *C. hepaticus* successfully colonizes the bile. Down regulation of flagella has also been observed in *C. jejuni* growth within the gastrointestinal tract. It has been suggested that *Campylobacter* might shut down flagellum production to evade the host immune system (Stintzi et al., 2005). *C. jejuni* flagella are required to pass the gastrointestinal tract of chickens but not for survival and persistence within the caeca (Wösten et al., 2004).

It is not currently possible to test the identified potential virulence genes, as the appropriate genetic tools for *C. hepaticus* have not yet been developed. However, this study adds a significant number of candidate gene targets for knockout and virulence-association assays due to the bioinformatics analyses performed. Utilizing the comparative genome approach, we have reduced the potential number of essential virulence genes from 1,709 to 1,059 and further in-depth genetic analysis has allowed us to generate a shortlist of likely virulence-associated genes (Table 2).

In conclusion, the *in vivo* transcriptome pattern of *C. hepaticus* found in this study was consistent with the nutrient-limited environment in bile. *C. hepaticus* harbors a wide range of potential virulence factors which we have identified using a comparative genomics and transcriptomics study. It appears that some of these genes play a key role in pathogenicity and adaptation of *C. hepaticus* to the low energy, low nutrient environments in chickens; in particular, gene clusters associated with glucose utilization, stress response, hydrogen metabolism and sialic acid biosynthesis. The virulence mechanisms that lead to the formation of liver lesions, mortalities and reduction in production in infected birds are yet to be elucidated but now a series of genes potentially involved in these processes have been identified.

## Data Availability Statement

All genomic assemblies and read sets have been deposited at NCBI (Bioproject PRJNA485661). The closed *C. hepaticus* HV10 genome has accession number CP031611.1. The raw RNA-Seq data and PacBio long-read DNA data were submitted to NCBI and can be accessed with accession number SAMN04544305.

## Author Contributions

RM, TV, and PS conceived and designed the experiments. TV, AA, and CP performed the experiments. TV, JL, and BV analyzed the data. TV and RM interpreted the data. TV, JL, and RM drafted the manuscript. All of the authors read and approved the final manuscript.

### Conflict of Interest Statement

AA and PS were employed by company Scolexia Pty Ltd. The remaining authors declare that the research was conducted in the absence of any commercial or financial relationships that could be construed as a potential conflict of interest.
